# A systematic review of botulinum toxin as a treatment for Raynaud’s disease secondary to scleroderma

**DOI:** 10.1007/s10067-024-07237-3

**Published:** 2024-11-30

**Authors:** Calver Pang, Despina Iakovou, Danny Fraser, Baptiste Leurent, Laura Awad, Benjamin Langridge, Peter Butler

**Affiliations:** 1 Department of Surgical Biotechnology, Division of Surgery & Interventional Science, Faculty of Medical Sciences, University College London, London, UK; 2 Department of Plastic and Reconstructive Surgery, Royal Free London NHS Foundation Trust, London, UK; 3 West Suffolk NHS Foundation Trust, Suffolk, UK; 4 Department of Statistical Science, University College London, London, UK

**Keywords:** Botulinum toxin, Raynaud’s disease, Scleroderma, Systematic review

## Abstract

Raynaud’s phenomenon (RP) is a vasospastic disorder that affects the small blood vessels in the extremities such as the hands, feet, fingers or toes. It is a debilitating condition that can severely impact the patient’s quality of life. Botulinum toxin (BTX) has been examined as a treatment option for RP, but its effect has been inconclusive. A systematic review has been conducted to determine the current evidence of BTX as a treatment for RP secondary to scleroderma. Major clinical databases Medline, Embase (via Ovid), the Cochrane Central Library, ClinicalTrials.gov, EU Clinical Trials Register and the ISRCTN registry were systematically searched from its inception to 27 November 2023 for studies describing BTX and RP. Standard mean differences of Quick-DASH scores, visual analogue scale pain (VAS-P) score and Raynaud’s condition score (RCS) are reported with BTX treatment with a random-effect model. A total of 890 entries were retrieved. Of these, 19 met the inclusion criteria, and all studies were included for analysis. There was a significant effect (*p* = 0.03) with Quick-DASH score and VAS-P score (*p* < 0.00001) but a non-significant effect (*p* = 0.37) with RCS. BTX is a therapeutic option in the treatment of RP secondary to scleroderma; however, the evidence published so far is not sufficient to credit it as a revolutionary first line treatment. More research is needed to establish dosing, techniques and core outcome measures for BTX in RP.

**Supplementary Information:**

The online version contains supplementary material available at 10.1007/s10067-024-07237-3.

## Introduction

Raynaud’s phenomenon (RP) is a common episodic reversible vasospastic disorder, which involves the arteries and arterioles of digits causing pain, pallor and paraesthesia secondary to triggers such as stress or cold weather [1]. It is a painful condition characterised by vasospasm where primary RP is a very common disorder affecting approximately 3–5% of the general population. In comparison, secondary RP can be particularly more severe and may lead to complications such as ulcers, scarring, and may require amputation of digits, and it can affect 18–46% of patients with systemic lupus erythematosus (SLE) and 97% of patients with systemic sclerosis [1, 2].

The primary cause of RP is unknown; however, it is often an early feature in connective tissue disorders. In these cases, there is a change in regulation of vasodilatory pathways to include nitrous oxide and calcitonin gene-related peptide. Secondary Raynaud’s is caused by a number of autoimmune connective disorders such as systemic sclerosis, SLE, Sjögren’s or mixed connective tissue disease. RP commonly represents the initial presenting symptom in patients with mixed connective disease [3, 4].

In secondary RP, where there are structural alterations in the vessel walls, the endothelial function is impaired. This leads to an imbalance between vasoconstriction and vasodilation and a subsequent mismatch between endothelium-derived vasoconstrictors such as endothelin-1 and vasodilators such as nitrous oxide and prostacyclin. RP patients also show impairments in neural regulation of vascular tone due to deficiency in calcitonin gene-related peptide contributing to impaired vasodilation. Intravascular abnormalities contributing to vasoconstriction include platelet activation, defective fibrinolysis, white blood cell activation, decreased red blood cell deformability and increase viscosity and oxidative stress. The underlying pathophysiology of RP involves a complex combination of both neural and vascular effects [3–6].

Vasodilatory therapy has shown to be effective in management of secondary RP with the first line agent being dihydropyridine calcium channel blockers. However, due to the lack of a single targetable pathway, the aetiology of RP treatment is limited, often involving multi-modal agents, which carry a greater risk of side effects [7–10]. Surgical management which includes amputation, hand stripping, nerve stimulation and fat grafting has variable efficacy and risk complications such as poor cosmesis, neuropraxia, paralysis and fat necrosis, respectively [5, 11].

To fill that gap in standardised and efficient treatment of RP, clinicians have been examining the effects of botulinum toxins (BTX) as a solution to the RP problem. BTX are an example of neurotoxins produced by *Clostridium difficile*; its mechanism of action in relation to its use in RP is hypothesised to be via the antagonism of vasoconstriction in arterioles of the digits secondary to blockade of the noradrenaline-mediated sympathetic pathway causing vasodilation alleviating symptoms of RP [12]. The use of botulinum toxin for treatment of RP was first utilised in 2004, since then there has been several studies suggesting it could be effective in the management of RP; however, not all studies found conclusive evidence.

This study aims to review the literature to determine the current evidence base for the efficacy of BTX as a treatment for RP secondary to scleroderma.

## Methods

The protocol for this review was registered on PROSPERO (CRD42023365143), and it is reported here following the Preferred Reporting Items for Systematic reviews and Meta-analysis (PRISMA) guidelines [13].

### Search strategy

The search was conducted on the following databases: Medline (via Ovid), Embase (via Ovid), Cochrane Library, clinicaltrials.gov, EU Clinical Trials Register and the ISRCTN registry, papers from the databse were searched from their inception to 27th November 2023. There were no language or date restrictions applied. An electronic literature search was conducted using free-text search terms: Botox, botulinum, toxin, onabotulinum, Raynaud*, scleroderma and systemic sclerosis. Combined with Boolean logical operators (see Supplementary Appendix 1 for the full search strategy). A search of the gray literature was performed by reviewing the reference list of papers included in the review and manually screening the reference list of related published systematic reviews.

### Study selection and data extraction

Study selection by title and abstract screening was performed by two reviewers (CP, DI) independently; any disagreements or discrepancies in any part of the process were resolved through discussion with a third reviewer (DF). Appropriate citations were then selected for full-text evaluation. Studies were included in the review if they assessed any clinical outcomes of botulinum toxin treatment. Case reports, letters, in vitro and animal studies were excluded. Studies included could be randomised trials, or observational studies, as long as reporting clinical outcomes with BTX compared to without BTX.

Data were extracted on study design, participant demographic characteristics, type of botulinum toxin treatment used, method of injection, the comparator population and clinical outcomes (incidence of clinical symptoms and Raynaud’s characteristics). Data were extracted on a pre-determined standardised form independently by three reviewers (CP, DI and DF) with cross checking after extraction.

### Quality assessment

Appraisal of the quality of the included studies was conducted by two reviewers independently. The randomised controlled trials were assessed for risk of bias by using the modified Jadad scale [14]. MINORS was used for risk of bias assessment in non-randomised studies[15]. The included case series were assessed using the JBI Critical Appraisal Checklist for Case series [16].

### Statistical analysis

For this systematic review, we selected outcomes commonly presenting in most of the included studies, visual analogue scale pain (VAS-P) score and Quick-DASH (Disabilities of the Arm, Shoulder, and Hand). Only patients receiving BTX injections were analysed, and no sub-group analysis was performed as there were varying methods of injections that we could not control for. Data synthesis and analysis were performed using dichotomous outcomes retrieved from the studies included, using a random effects model. However, due to the single arm studies, we calculated for standard means change of these outcomes, looking at the difference before and after injections in single arm studies. The pooled results were presented as risk rations with 95% confidence intervals (CI). *I*^2^ statistics were used to assess heterogeneity. A *I*^2^ value of more than 75% was considered suggestive of substantial heterogeneity. For this meta-analysis, we used Review Manager 5.4 software.

## Results

### Study characteristics

**Fig. 1:**
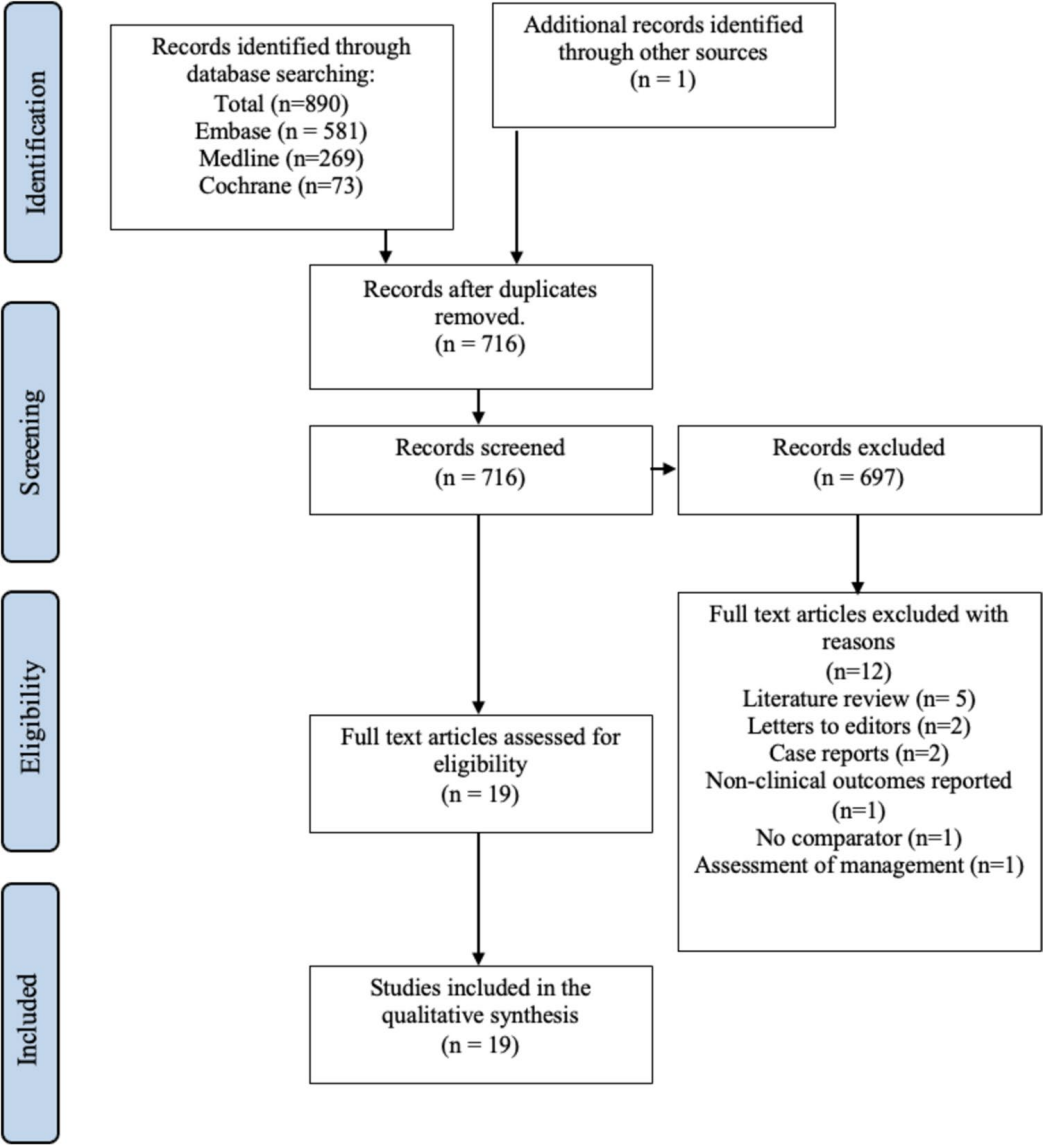
PRISMA chart

The study selection process and the results of the literature search are presented in Fig. 1. The search identified 890 abstracts, and 19 eligible studies were included in the review. The main characteristics, including the type of study and the participant demographics, are presented in Table 1. All articles were published in the English language, between the years 2007 and 2022. The study population size is ranging from 2 to 91 participants. There were six randomised double blind controlled trials, seven case series (*n* ≥ 2), two retrospective chart reviews and four cohort studies. Part of the included studies include both male and female participants, whereas the majority do not specify. One study used only female participants.

**Table 1:** Patient demographics and study characteristics

Authors	Type of study	Study population	Mean age (years)	Limited/diffuse scleroderma	Sex
Bello [17]	Randomized, double blind, parallel-group, placebo, clinical trial	40	52	25/15	Female: 31 (78%) Male: 9 (22%)
Dhaliwal [18]	Prospective case series	40	48	20/20	Female: 40 (100%)
Du W [19]	Randomized self-control clinical trial	16	44.8	8/8	Female: 16 (100%)
Fregene [20]	Retrospective chart review	26	55	Not stated	Female: 14 (54%) Male: 12 (46%)
Goldberg [21]	Retrospective cohort study	20	53	Not stated	Female: 13 (65%) Male: 7 (35%)
Habib [22]	Case series	3	Case 1: 50 Case 2: 25 Case 3: 23	Not stated	Female: 3 cases (100%)
Medina [23]	3-year retrospective cohort study	15	46	7/2	Female: 14 (93%) Male: 1 (7%)
Motegi [24]	Prospective, single-blind, randomized, investigator-initiated, clinical trial	45	61	26/19	Female: 41(91.1%) Male: 4 (8.9%)
Motegi [25]	Prospective, case series study	10	63	4/6	Female: 7 (70%) Male: 3 (30%)
Nagarajan [26]	Retrospective observational study	11	53	Not stated	Female: 10 (90.9%) Male: 1 (9.1%)
Quintana Castanedo [27]	Single-centre prospective study (case series)	8	16	Not stated	Female: 6 (75%) Male: 2 (25%)
Senet [28]	Randomized, double-blind, placebo-controlled multicentre study	90	54	65/16	Female: 80 (72%) Male: 10 (28%)
Seyedmardani [29]	Double-blind randomized controlled trial	11	46	7/4	Female: 11 (100%)
Shenavandeh [30]	Clinical trial	16	Not stated	20/6	Female: 11 (68.7%) Male: 5 (31.2%)
Uppal [31]	Prospective cohort study	20	37	Not stated	Female: 20 (100%)
Van Beek [32]	Case series	11	51	Not stated	Female: 9 (82%) Male: 2 (18%)
Winter [33]	Case series	4	Case 1: 68 Case 2: 39 Case 3: 42 Case 4: 65	2/0	Female: 4 (100%)
Zhang [34]	Retrospective cohort study	10	61	Not stated	Female: 5 (50%) Male: 5 (50%)
Zhao [35]	Case series	2	Case 1: 34 Case 2: 41	Not stated	Female: 2 (100%)

As per the inclusion criteria, all studies included participants with RP secondary to scleroderma as the main diagnosis, as well as all of them mentioned similar vocabulary to describe the severity of disease for all patients that took part in the studies.

### Treatment and comparator

All botulinum toxin doses and anatomical injection sites are presented in Table 2. There was no standardised way of injecting between the included studies. The research studies differed with respect to doses and sites of BTX, with a minimum of 20 and the maximum of 300 units per hand. This was due to studies using either BTX-A or BTX-B, where the higher doses were accounted for by the later. The location of the injection sites varied; a few studies described the locations, commonly between palmar aspect and dorsal aspect. Two studies specified in detail the units of BTX-A used in each finger.

**Table 2:** Botulinum toxin dose and anatomical injection site

Authors	Botox dose (units)	Control	Injection site
Bello [17]	50 units per hand	Sterile saline in opposite hand. Total 2.5 ml	Digital neurovascular bundle—dorsal approach
Dhaliwal [18]	100 units across both hands	N/A	Digital neurovascular bundle—dorsal approach
Du [19]	20 units per hand	Nil	Neurovascular plexus at the metacarpophalangeal level of the second-third and third-fourth fingers
Fregene [20]	Average of 92 units per treatment course	N/A	Base of proximal phalanx—palmar approach Distal palm at level of superficial palmar arch Proximal hand at level of distal volar wrist crease
Goldberg [21]	100 units per hand	N/A	Perivascular space on the palmar surface and at the wrist
Habib [22]	64 units across both hands	N/A	Web space of digits 2 to 5 on both hands near the metacarpophalangeal joints
Medina [23]	Average of 42.8 units for left hand and average of 47.3 units for right hand	N/A	Base of the lateral aspects of all fingers, except the first
Motegi [24]	BTX-B: 250 units (*n* = 9), 1000 units (*n* = 10) and 2000 units (*n* = 18) per hand	Nil	Palmar aspect of the hand, just proximal to the A1 pulley
Motegi [25]	10 units into one finger with most severe symptoms	Nil	Palmar aspect of the hand, just proximal to the A1 pulley
Nagarajan [26]	156 units (average) across both hands	N/A	Base of the digit around palmar digital neurovascular bundle and radial and ulnar artery at wrist level for severe ischaemic symptoms and digital ulcers
Quintana Castanedo [27]	36 units per hand	N/A	Dorsal approach into each finger webspace and each side of the thumb and fifth finger’s metacarpophalangeal joint
Senet [28]	50 units per hand	0.9% saline	Distal palmar of each hand, targeting neurovascular bundles in the 4 web spaces
Seyedmardani [29]	50 units per hand	0.9% saline	Dorsal approach to webspace of digits and
Shenavandeh [30]	20 units per finger	Abobotulinumtoxin-A, 20 units for each affected finger	Medial and lateral sides of the root of every involved digit
Uppal [31]	100 units per hand	N/A	At level of distal palmar crease in the web spaces around the digital neurovascular bundles of all five digits
Van Beek [32]	100 units per hand	N/A	Digital vessels to all fingers except thumb unless it is symptomatic
Winter [33]	Abobotulinumtoxin-A 60–300 units per finger or hand	N/A	Distal palmar crease between the metacarpals and webspace between digits
Zhang [34]	50 units per hand	N/A	Palmar approach around neurovascular bundles at the level of the metacarpophalangeal joint, and para-ulnar artery and para-radial artery
Zhao [35]	Case 1: 200 units across both hands Case 2: 280 units across both hands	N/A	Palmar approach along the track of the digital vessels of each finger and palm

The comparator used in each study was the other hand than the one with the Botox injection or the condition prior to the injections.

### Outcomes

All outcomes reported in the included studies are presented in Table 3.

**Table 3:** Outcomes and follow-up

Authors	Primary outcome results	secondary outcome results	Follow-up
Bello RJ 2017 [17]	The absolute blood flow at 1-month follow-up in BTX-A hands was significantly lower than placebo (*p* = 0.018)	Changes in QuickDASH scores were not significantly different between arms at 1-month (*p* = 0.504) or 4-month follow-up (*p* = 0.388)	1 month and 4 months
		McCabe cold sensitivity scores in BTX-A hands were not significantly different at 1-month (*p* = 0.834) or 4-month follow-up (*p* = 0.963)	
		VAS pain score was not significantly different at 1-month (*p* = 0.121) or 4-month follow-up (*p* = 0.585)	
		Oxygen saturation was not significantly different at 1-month (*p* = 0.318) or 4-month follow-up (*p* = 0.074)	
		Patient-reported RCS was statistically different (*p* = 0.045)	
Dhaliwal K 2019 [18]	At 6 weeks, there was a significant improvement in DASH score (*p* = 0.001) and pain (*p* = 0.001)	N/A	6 and 12 weeks
	At 12 weeks, there was a significant improvement in DASH score and pain (*p* < 0.05)		
	At 6 weeks, mean hand strength increased in both dominant and non-dominant hand post injection (*p* < 0.05) Kapandji score decreased in both dominant hand (*p* = 0.001) and non-dominant hand (*p* < 0.05)		
	At 12 weeks, mean hand strength, Kapandji score and ROM significantly improved (*p* < 0.05)		
	At 6 weeks, 88% reported improvement in symptoms including reduction in pain, improved color change and reduced swelling. Of these patients, 80% reported an improvement in cold intolerance with reduction in frequency and severity of Raynaud's attacks over the first 6 weeks. By 12 weeks, 70% of patients reported an improvement in symptoms including pain reduction, improved colour change and reduced swelling		
	At 6 weeks there was a mean increase in temperature of the index to little fingers (*p* < 0.05) but at 12 weeks no change in thermographic readings		
Du W 2022 [19]	Reynold’s score (*p* < 0.001) and temperature change (*p* < 0.001) improved with BTX-A	N/A	4 weeks
	VAS pain score, Quick-DASH, mRSS, digital ulcer score was not significantly different after treatment.		
	Dermoscopic parameters (*p* = 0.002), nailfold capillary pattern staging (*p* = 0.004) improved with BTX-A fingertip baseline skin temperature, skin temperature recovery after cold water stimulation, digital ulcer score, and the dermoscopic qualitative37 and semiquantitative stages38 were evaluated and recorded.		
Fregene A 2009 [20]	Statistically significant improvements for VAS pain score (*p* < 0.01) and digit transcutaneous oxygen saturation measurements after treatment (*p* < 0.05)	Determine injection pattern success between distal palm and digit injection. Tendency towards palm injections but not statistically different (*p* = 0.57)	18 months
	No significant difference between colour change (*p* = 0.44)		
	11 out of 23 ulcers healed after the treatment course completion.		
Goldberg SH 2021 [21]	11 out of 13 patients who had 20-minutes post treatment scores had pain decreased on the NRS-11 or VAS pain score and was sustained for 3 months	Median morphine equivalent usage view decreased from 82.5 to 0 after injection.	Average follow-up of 10.5 months
	QuickDASH scores demonstrated clinically significant improvements at 6 weeks, 3 months, and 6 months		
Habib SM 2020 [22]	Pain: - patient 1: from 8 to 0 -patient 2: no effect after 1 and 2-weeks follow-up - patient 3: free of symptoms	N/A	1 week for patients 1,2,3 Patient 2 also had 2-week follow-up
Medina 2018 [23]	Statistically significant improvements for VAS pain score (*p* < 0.005) and number of episode of Raynaud’s phenomenon (*p* < 0.009) after one week	N/A	2–3 years
	5 out 7 patients with basal ulceration completely healed after 3 months		
Motegi Si 2017 [24]	Raynaud’s scores in group treated with 250 units was significantly lower than control group (*p* < 0.05) and groups treated with 1000 and 2000 units were significantly lower than control group and 250-unit group (*p* < 0.01) at 4 weeks	N/A	4, 8, 12, 16 weeks
	VAS pain score in groups treated with 1000 and 2000 units were significantly lower than in the control group at 4 weeks (*p* < 0.01)		
	Skin temperature recovery after cold-water simulation was significantly improved (*p* < 0.01) in group treated with 2000 units compared with control group and group treated with 250 units.		
	Digital ulcers treated with 1000 units and 2000 units were significantly different at 4 weeks (*p* < 0.05) compared to control and 250 units group		
Motegi Si 2016 [25]	Raynaud’s score improved significantly at 4 weeks (*p* < 0.05)	Skin temperature recovery after cold water simulation improved significantly at 4 weeks (*p* < 0.05)	2, 4, 8, 12, 16 weeks
	VAS pain score improved significantly at 4 weeks (*p* < 0.01)	5 patients with digital ulcers all healeed by 16 weeks	
		Raynaud’s score improved significantly at 8, 12 and 16 weeks (*p* < 0.01)	
		VAS pain score improved significantly at 2 weeks (*p* < 0.05) and 16 weeks (*p* < 0.01)	
Nagarajan 2021 [26]	All patients reported an improvement in symptoms and healing of digital ulcers		Mean 49 months
Quintana Castanedo L 2021 [27]	The mean VAS score for pain was reduced from 7.5 to 0.8 points	6 patients reported a reduction in monthly RP episodes.	1 month
		Mean reduction on the cold sensitivity severity scale was 15 points	
Senet P 2022 [28]	No statistical difference in median numbers of daily RP attacks at 4 weeks (*p* = 0.77)	No statistical difference in median numbers of daily RP attacks at 12 and 24 weeks (*p* > 0.4)	4, 12, 24 weeks
	Number of RP attacks reduced by 22% at week 4.	No statistical difference between in RCS, HAQ-DI-assessed quality of life, QuickDASH and CHFS-evaluated hand function at 4, 12 and 24 weeks.	
		VAS pain score decreased by 24% at 4 weeks with BTX-A	
		BTX-A group experienced transient hand-muscle weakness significantly more frequently (*p* = 0.003)	
Seyedmardani 2021 [29]	No statistically significant difference for frequency of new ulcer formation at one month (*p* = 1.00) and two months (*p* = 0.48)	Statistical difference at two months in recurrence Raynaud’s attacks interval (*p* < 0.01), pain relief (*p* < 0.01), change of skin colour (p*p* < 0.01) and duration of Raynaud’s (*p* < 0.01)	1 and 2 months
	Significant improvement in RCS at two months (*p* < 0.01), Quick-DASH score at two months (*p* < 0.01), McCabe CSS at one (*p* < 0.001) and two months (*p* < 0.01).	No statistical difference in frequency of complications (*p* = 0.148)	
	No statistical difference in VAS pain score at one (*p* = 0.46) and two months (*p* = 0.09)		
Shenavandeh S 2022 [30]	Statistical difference in digital ulcers healed (*p* < 0.0001)	Capillaroscopy: -Haemorrhage disappeared significantly (*p* = 0.03) -No significant changes in capillary distribution, morphology, dimension, density, presence or elongation, avascular area, and abnormal shapes (*p* > 0.05)	1 month
	Statistical difference in VAS pain score (*p* < 0.0001)		
	No statistical difference on digital ischaemia and RP (*p* > 0.05)		
Uppal L 2014 [31]	Statistical difference in hand function improvement: pure pinch (*p* = 0.05), tripod pinch (*p* = 0.01), and power grip (*p* = 0.05)	N/A	8–12 weeks
	Statistical difference in improvement in range of movement of index (*p* = 0.03) and middle (*p* = 0.06) finger metacarpophalangeal joint flexion		
	80% improvement in VAS pain score and DASH score, 75% improvement in colour and 65% improvement in cold intolerance but not statistically significant		
Van Beek AL 2007 [32]	All participants had pain relief of digital rest pain from 9-10 of 10 to a level of 2 of 10.	Surface temperatures from 1.0°C to 4.0°C increases in surface temperature	Average 9.6 months
Winter AR 2020 [33]	Case 1: Improved skin quality at 9 and 12 months	N/A	9, 12, 14, 17, 21 months
	Case 2: VAS score pain improved from 3.5/10 to 1/10 at 12 months		
	Case 3: Improved pain from 8/10 to 2/10 within a week		
	Case 4: Improved pain and skin induration for 13 months		
Zhang 2015 [34]	Statistical difference artery flow velocity (*p* < 0.01), surface temperature (*p* < 0.01) and VAS pain score (*p* < 0.01)	N/A	Average 6 months
Zhao and Lian 2015 [35]	Case 1: VAS of cold sensation reduced from 8 to 2 after two days	N/A	5–6 months
	VAS pain score reduced from 6 to 0 and VAS stiffness reduced from 7 to 2 after two weeks		
	Case 2: VAS stiffness reduced from 6 to 3, improved colour change and improved skin temperature from 23.3 ^o^C to 27.9^o^C at one week		

Pain assessed by the VAS score was the most common outcome, mentioned in 18 studies. Six studies showed statistically significant change in the VAS-P score comparing pre- and post-injection values or BTX-A versus placebo values. DASH and Quick-DASH scores were used to measure upper extremity function in seven studies. Five used Quick-DASH (11 questions) and two used DASH (30 questions), of which one study from each respective outcome measure tool showed statistical improvement. The Quick-DASH questionnaire is a validated and widely used tool that measures upper-extremity specific symptoms and disability [36].

The Raynaud’s condition score (RCS) is another tool used to quantify the severity of disease and is classed as a patient reporting outcome tool. Four studies used the RCS, and all had statistically significant improvement in the results. In addition to the RCS, the crude number of episodes before and after the treatment was also measured. Two studies showed a statistically significant change in the number of episodes.

Difference in skin temperature was also a frequently reported outcome found in seven studies. Four of those studies reported a statistically significant result. Temperature, along with the frequency of ulcer healing post-treatment, was not widely reported. Seven studies reported ulcer healing as an outcome; only one of them reported a statistically significant increase in healing frequency.

## Quality assessment

Table 4 summarises the results of risk of bias assessment. According to the modified Jadad scale, six randomised controlled trials were of high quality, with all scoring 6 points or more. Four studies scored at least 12 points through the MINORS index. Five studies scored at least 12 points or more through the adjusted Newcastle–Ottawa Scale quality assessment.

**Table 4:** Quality assessment summary

**JBI critical appraisal checklist**												
Author	Type	Q1	Q2	Q3	Q4	Q5	Q6	Q7	Q8	Q9	Q10	Overall appraisal
Habib 2020	Case series	No	No	No	Yes	Unclear	Unclear	Unclear	No	No	No	Exclude
Van Beek 2007	Case series	Yes	Yes	Unclear	Unclear	Yes	Yes	Unclear	Yes	No	No	Exclude
Winter 2020	Case series	No	Unclear	Unclear	Unclear	Unclear	Yes	No	No	No	No	Exclude
Zhao 2015	Case series	No	Unclear	Unclear	Unclear	Unclear	No	No	No	No	No	Exclude
**MINORS**												
Author	Type	I	II	III	IV	V	VI	VII				
Quintana Castanedo 2021	Single-centre prospective study (case series)	2	2	2	1	1	2	2				
Uppal 2014	Prospective cohort study	2	2	2	1	2	2	2				
Dhaliwal 2019	Prospective case series	2	2	2	1	2	2	2				
Motegi 2016	Prospective, case series study	2	2	2	2	1	2	2				
**Adjusted NOS quality assessment**												
Author	Type	Q1	Q2	Q3	Q4	Q5	Q6	Q7				
Goldberg 2021	Retrospective cohort study	2	2	2	2	2	2	2				
Fregene 2009	Retrospective cohort study	2	2	2	2	2	2	2				
Nagarajan 2021	Retrospective cohort study	2	2	1	2	2	1	2				
Medina 2018	Retrospective cohort study	2	2	1	2	1	2	2				
Zhang 2015	Retrospective cohort study	2	1	2	2	2	2	2				
**Modified Jadad scale**												
Author	Type	I1	I2	I3	I4	I5	I6	I7	I8	Total		
Shenavandeh 2022	Clinical trial	1	1	1	1	1	1	0	1	7		
Seyedmardani 2022	Double-blind randomised controlled trial	1	1	1	1	1	1	0	1	7		
Du 2022	Randomized self-control clinical trial	1	1	1	1	1	1	0	1	7		
Bello 2017	Randomized, double blind, parallel-group, placebo, clinical trial	1	1	1	1	1	1	0	1	7		
Senet 2022	Randomized, double-blind, placebo-controlled multicentre study	1	1	1	1	1	1	1	1	8		
Motegi 2017	Prospective, single-blind, randomized, investigator-initiated, clinical trial	1	1	1	0	0	1	1	1	6		

### Meta-analysis

**Fig. 2:**
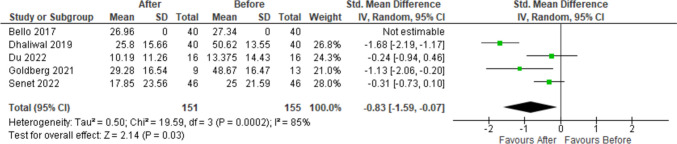
Forest plot of the meta-analysis for Quick-DASH score

**Fig. 3:**
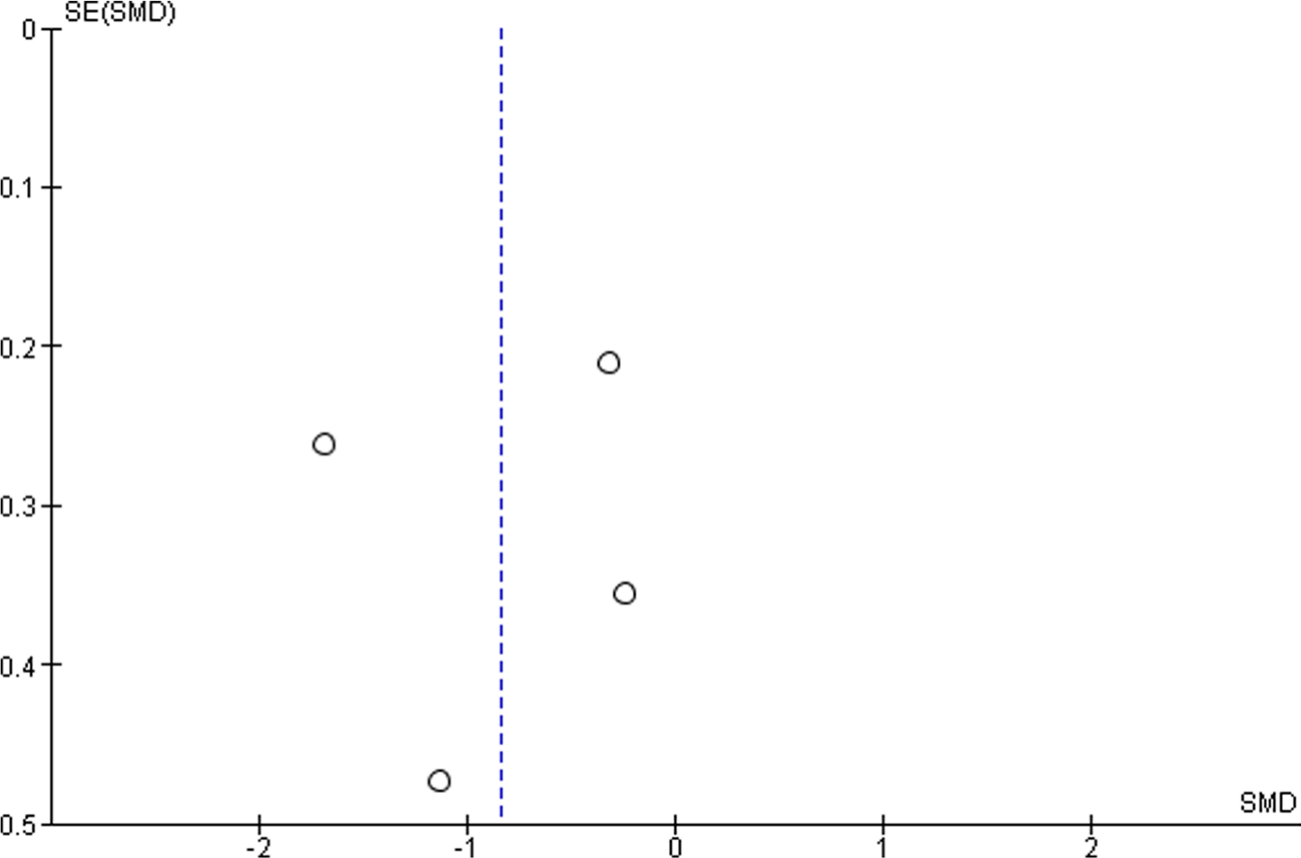
Funnel plot for the Quick-DASH scores

For a single-arm meta-analysis of the Quick-DASH scores, five research papers were included, and out of them, only four have all the mean and standard deviation (SD) of the Quick-DASH scores reported before and after the BTX-injection. In total, there are 155 patients before the treatment and 151 patients after the treatment. Overall mean Quick-DASH score is significantly reduced by 0.83 with 95% C.I. given by (− 1.59, − 0.07) (Fig. 2). The largest reduction is reported by Dhaliwal et al. [18] with a reduction of 1.68 in the standardised score. To compute the overall effect, we have used a random effects model, and there is a significant effect with *p*-value = 0.03. However, the heterogeneity in the reported results is also significant (*p*-value = 0.0002). The funnel plot in Fig. 3 does not show any significant publication bias; however, the sample size is too small to have any meaningful statistical reasoning.

**Fig. 4:**
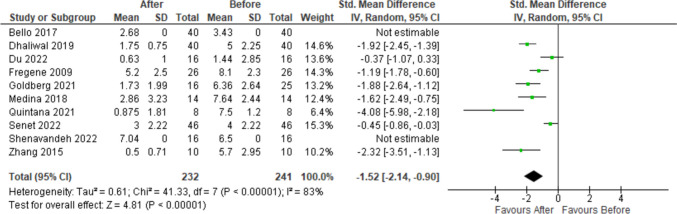
Forest plot of the meta-analysis for VAS-P score

**Fig. 5:**
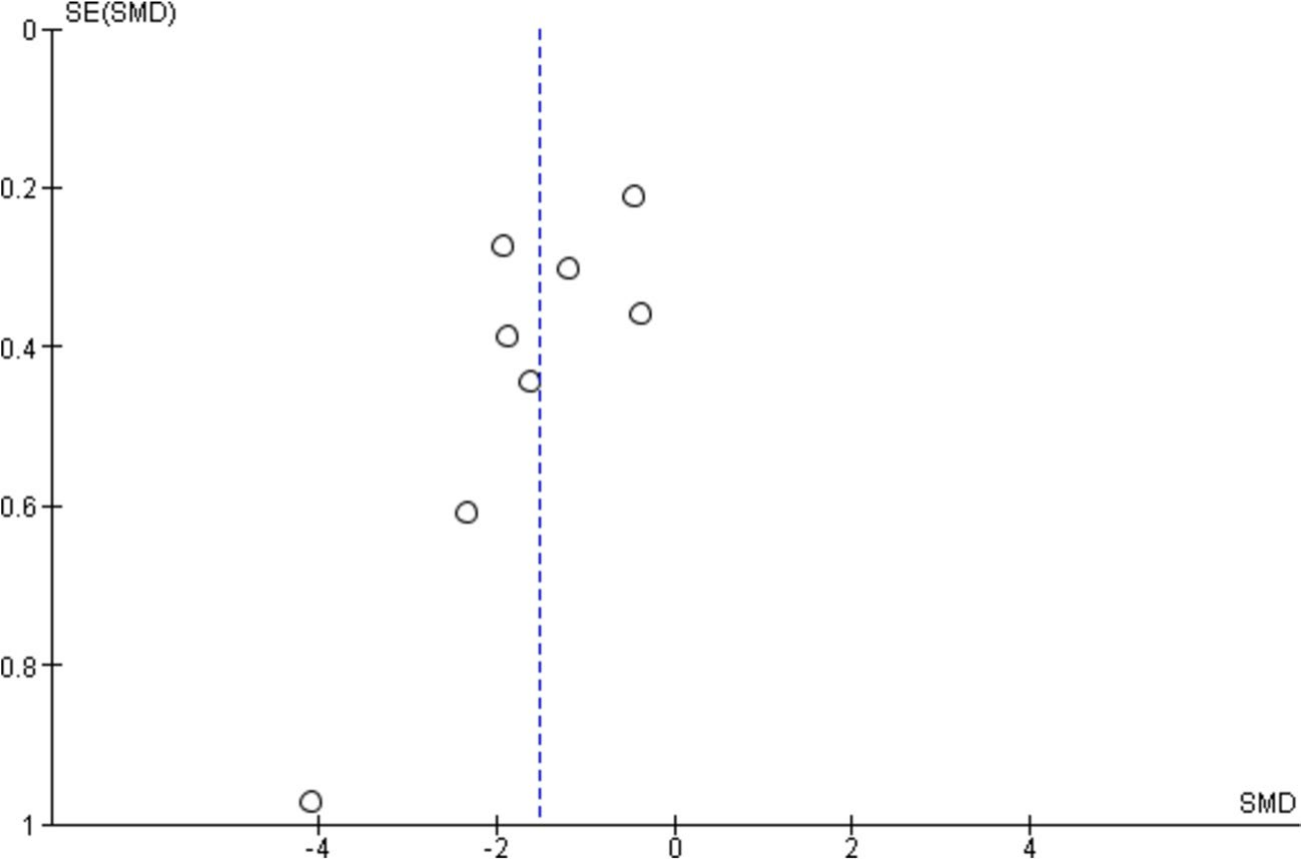
Funnel plot for the VAS-P scores

**Fig. 6:**
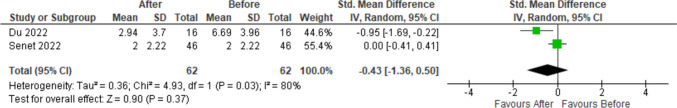
Forest plot of the meta-analysis for RCS

**Fig. 7:**
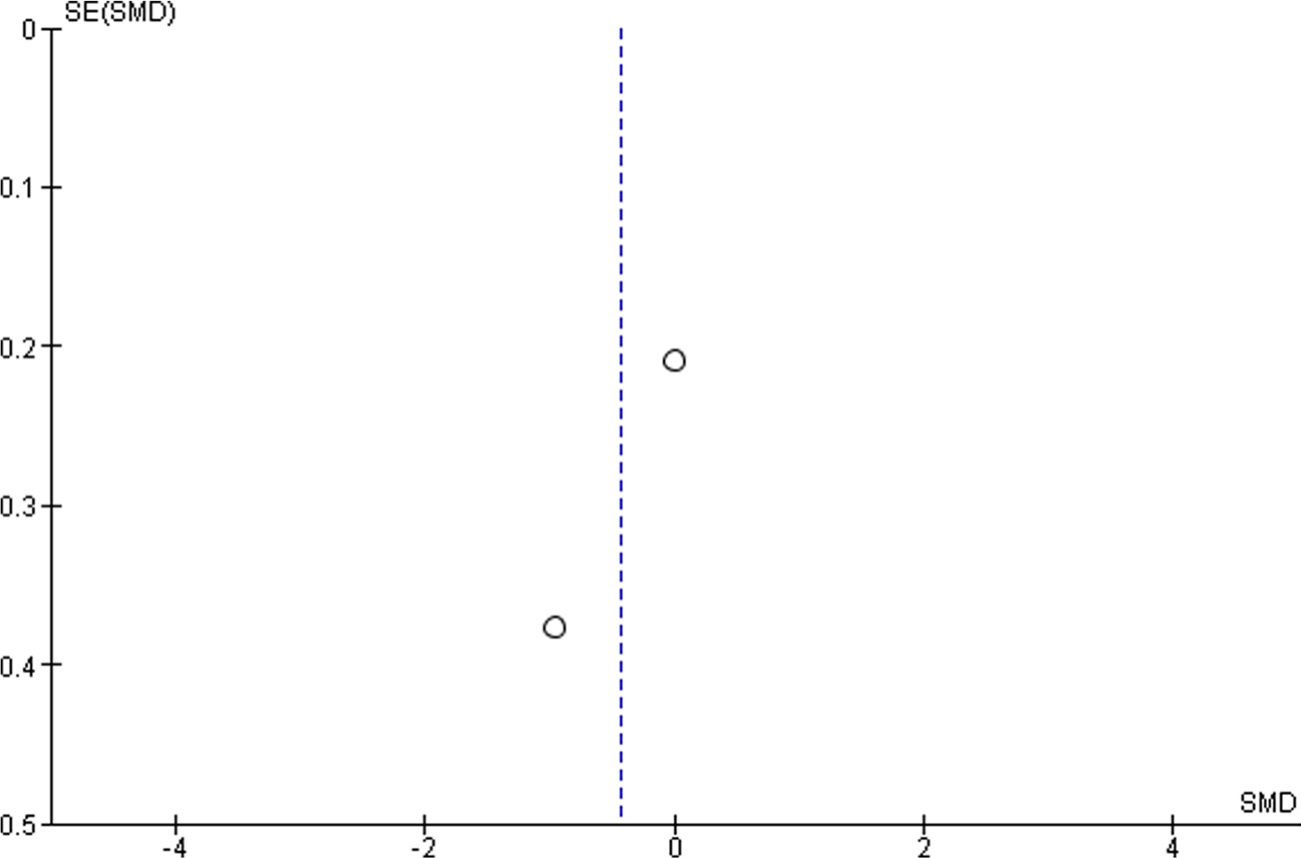
Funnel plot for the RCS

Figure 4 shows the forest plot of the meta-analysis of VAS-P score from ten studies with eight studies having the complete information. Total number of patients analysed before the treatment is 241 and after the treatment is 232. Except for Du et al. [19], all others reported significant decrease in VAS-P score, and the overall standardised mean VAS-P score is also reduced by 1.52 with a 95% C.I. of (− 2.14, − 0.90). The overall effect of the treatment is also significant with *p*-value < 0.00001. The heterogeneity of the reported results is also significant (*p*-value < 0.000001). The funnel plot in Fig. 5 does not show any significant departure from symmetry, and hence, there is unlikely to be any publication bias. Figure 6 shows the forest plot of the meta-analysis of RCS from two studies, with a total of 62 patients reported. There is no significant reduction RCS with a 95% confidence interval of (− 1.36, 0.50). The overall effect has a *p*-value of 0.37, which also shows that there is no significant effect of the treatment on RCS. The funnel plot is reported in Fig. 7 and with only two observations; this is inconclusive.

## Discussion

To our knowledge, this is the third systematic review examining the clinical outcomes of patients with RP secondary to scleroderma, treated with BTX injections.

The studies included in this review vary greatly in design and methodology. The population of all studies included small groups of patients, and each study used dissimilar areas of injection as well as various volumes of treatment injections. The population was not well described in a majority of study.

BTX does not have significant interactions with medications besides medication that have neuromuscular blocking effects such as pancuronium and antibiotics such as neomycin. Thus, it is considered safe to combine local BTX with medications such as prostacyclins and PDE-5 inhibitors [37].

Local BTX is often considered when lifestyle modifications and pharmacological therapies are unsuccessful. Van Beek et al. [32] first reported the use of BTX-A in the setting of connective tissue disease demonstrating symptomatic improvement and no structural vaso-occlusive disease proximal to the wrist, and ulcer healing in eleven patients and nine patients with RP, respectively. Like Van Beek et al., there have been multiple studies focusing on the most severe complication of secondary RP, digital ulcers. Clinical trials like Jokar et al. have managed to show its efficiency and its beneficial therapeutic effect; however, the mechanism of action is unknown, and there have been no statistically significant findings in our review to demonstrate the positive effect of BTX in digital ulcers [38]. Since then, further studies have investigated the effectiveness of BTX in secondary Raynaud’s. However, the results have been varied, with different doses and techniques used by authors, and thereby, no one agreed standardised approach.

Despite the use of various injection protocols, all studies target specific neurovasculature for each finger and the palm. The injection sites were not described in each study and were described differently in each case. However, the majority of the studies injected BTX at finger webspace to target the neurovascular bundle with an equal distribution between palmar and dorsal approach. Most studies did not specify the thought process behind each injection technique, and there has been insufficient evidence in the literature to suggest whether the therapeutic effect is location or dose-dependent. However, there has been reported complications of intrinsic hand muscle weakness associated with a palmar approach with rates as high as 27% [20, 31, 39–41] A dorsal approach will protect the lumbricals from the effects of BTX thereby reducing the risk of hand weakness. However, further research will be required to fully assess the significance and complications rates between the different injection approaches. As seen in this study by Figueroa et al. [42], it is possible to determine an optimal dose of BTX-A to establish treatment of RP; however, there have not been sufficient studies to determine this. The long-term BTX-A efficacy is yet to be determined in order to specify the need of repeat injections for RP, as BTX-A effect is known to decrease over time. This systematic review does not include studies with multiple repeat injections; however, this needs to be established to create treatment protocols to include BTX-A for RP in the future [23].

**Fig. 8:**
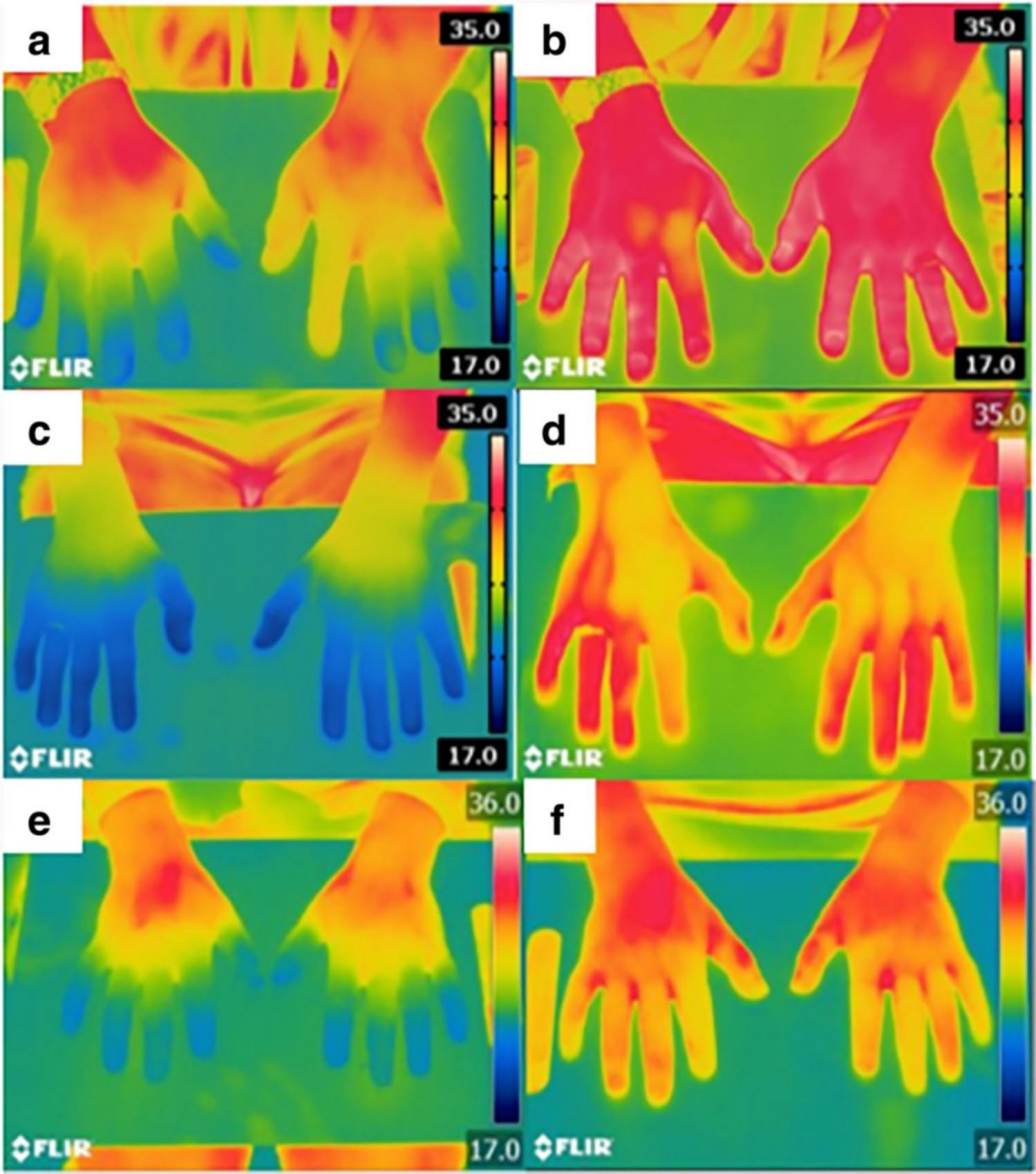
Thermographic imaging before and after Botox-A injections. Images demonstrate an increase in temperature (°C) from pre Botox-A (**a**, **c**, **e**) to post Botox-A (**b**, **d**, **f**) in the dominant and non-dominant hands at 6 weeks following 50 units of Botox-A via a dorsal approach. From Clinical Rheumatology, Springer Nature, by Dhaliwal (2019). Reprinted with permission

Our results suggest that BTX-A injections in the hand contribute to a statistically significant improvement in the clinical outcomes of pain, disability and strength, evaluated by the VAS-P and the DASH score. However, other less clinically relevant outcomes were also assessed and showed significant improvement in our studies such as skin temperature, showing an increase in skin temperature of the fingers affected by severe RP post-injections (Fig. 8). One study showed a significant increase in temperature, which was dependent on the geographical location of the patient [17].

Environmental and skin temperature play a major role in the manifestation of the disease as they constitute a trigger for disease flare up. A RP attack can be triggered by exposure to cold temperature or even mild temperature changes from warm to cooler. The normal response of blood vessels in response to change of ambient temperature is complex, and in RP that delicate system is altered [43].

Pain is a basic characteristic of RP along with colour changes (pallor, cyanosis and erythema), sensation of pins and needles and increased sensitivity of the digits affected. These symptoms are directly related to vascular abnormalities and the body’s impaired response to changes in temperature [6]. Studies by Seyedmardani et al. [29] and Zhang et al. [34] demonstrated a statistically significant reduction to pain at follow-up after BTX-A injections compared to the control group of each study, demonstrating the effects of BTX-A in improving blood flow and pain scores. Most studies included outcome measures such as VAS-P score and Quick-DASH; however, ASRAP (assessment of systemic sclerosis-associated Raynaud’s phenomenon) questionnaire has been developed and is an instrument that has been robustly developed for systemic sclerosis-associated RP. This would be a useful efficacy outcome to be included in assessing patients who receive BTX treatment.

The limitation of most studies was the exclusion of a control group thereby enabling no direct comparison. In addition, the duration of the studies were often limited to months of follow-up and thus unable to assess the long-term effects of BTX. The doses of BTX varied amongst each study with no study assessing titration of doses in attempt to optimise dosage. In addition, the varied injection points did not allow to a conclusion of an ideal injection approach. Nevertheless, the findings from this systematic review supports the efficacy and safety of BTX for the treatment of RP secondary to scleroderma.

BTX-A injections in managing RP secondary to scleroderma have the potential and might have already become a revolutionary solution to the complications caused by RP as it is also minimally invasive and has a minimal rate of compilations. Studies carried out by Neumeister et al. [44] demonstrate statistically significant efficacy of Botox improving perfusion and reduction of pain in the digits; there is no sufficient evidence to determine the mechanism of action which reflects on the pressing need for randomised control clinical trials with quantifiable PROMs such as the DASH score, to limit objectivity and increase reliability of the data.

## Conclusion

BTX-A is a therapeutic method shown to solve a significant problem for patients with RP secondary to scleroderma; however, the evidence published so far is not sufficient to credit it as a revolutionary first line treatment. Ongoing clinical trials such as the one in Emory University Hospital, Georgia, USA (NCT05125029), are needed to confirm the need for BTX-A in treatment of RP. As shown in this review, there is great diversity in the methodology of each study, and data evaluation is impaired. Patient-reported outcomes, however useful, can be objective and difficult to generalise and compare if not standardised. Each study measured most outcomes in different ways, making the development of core outcome sets necessary for future evaluation of the BTX-A use in Raynaud’s and BTX-A techniques.

## Supplementary Information

Below is the link to the electronic supplementary material.

Supplementary file1 (DOCX 14 KB)
