# Supplementary material for: A systematic review of botulinum toxin as a treatment for Raynaud’s disease secondary to scleroderma
Source: Clin Rheumatol. 2024 Nov 30;44(1):81–96. doi: 10.1007/s10067-024-07237-3 (PMC11729122; doi:10.1007/s10067-024-07237-3)
Supplement: Supplementary file 1 — Supplementary file1 (DOCX 14 KB) [file 10067_2024_7237_MOESM1_ESM.docx]

**Appendix 1:** Search Strategy

**Search Terms**

| 1. Botox or botulinum or toxin or onabotulinum |
| --- |
| 1. Raynaud* or Scleroderma or systemic sclerosis 2. 1 AND 2   Additional MeSH search: |
| 1. MeSH descriptor: [Botulinum Toxins, Type A] 2. MeSH descriptor: [Raynaud Disease] 3. 1 AND 2 |
